# Risk factors for uterovaginal prolapse among women in public hospitals of Sidama, Ethiopia: a case control study

**DOI:** 10.3389/frph.2025.1569449

**Published:** 2025-09-18

**Authors:** Hirut Yosef, Tsegaye Alemu, Mekdes Wondirad Mengesha, Aklilu Adule

**Affiliations:** ^1^ Reproductive Health Department, Yanet-Liyana College of Health Sciences, Hawassa, Ethiopia; ^2^ School of Public Health, College of Medicine and Health Sciences, Hawassa University, Hawassa, Ethiopia

**Keywords:** gynecological outpatient, risk factors, uterine prolapse, women, case control design, public hospitals

## Abstract

**Introduction:**

Utero-vaginal prolapse is a significant public health concern in developing countries such as Ethiopia, where access to health care is limited. It is a major reproductive crisis in women that affects a woman's quality of life and has a great negative impact on women's social, physical, economic, and psychological wellbeing. Despite this, there is limited evidence on risk factors in the study area. Therefore, this study aimed to identify the risk of utero-vaginal prolapse among women visiting gynecologic outpatient departments in governmental hospitals.

**Methods:**

A facility-based unmatched case–control study was conducted among 286 women visiting gynecologic outpatient departments in selected governmental hospitals. The data were collected via a pretested structured questionnaire designed with a Kobo tool box. The Kobo tool is an easy, open electronic data collection tool suitable for field research and helps ensure data security. The data were subsequently exported to SPSS for analysis. Descriptive statistics were performed. To assess associations, independent *t*-tests and binary and multivariate logistic regression analyses were performed. Finally, a 95% confidence interval and adjusted odds ratio with a *p* value <0.05 were used to examine the associations between the dependent and independent variables.

**Results:**

A total of 277 respondents, 91 patients with utero-vaginal prolapse and 186 controls, were included in the study. According to the multivariable logistic regression analysis, early childbirth [AOR = 3.98 (95% CI: 1.08–14.58)], a history of multiple pregnancies [AOR = 2.88 (95% CI: 1.27–6.49)], home delivery [AOR = 4.9 (95% CI: 1.3–18.6)], prior pelvic surgery [AOR = 3.9 (95% CI: 1.08–13.8)], and a history of instrumental delivery [AOR = 3.1 (95% CI: 1.08–9.14)] were found to be significant determinants of utero vaginal prolapse.

**Conclusion:**

These findings underscore that *in utero* vaginal prolapse is a common reproductive health problem. Early childbirth, a history of multiple pregnancies, home delivery, prior pelvic surgery, and a history of instrumental delivery were risk factors for UVP. Therefore, social and health care system determinants are critical. Therefore, prevention of UVP requires promoting health facility deliveries, integrating obstetric care, and addressing the societal norms that may lead to early childbirth. Consequently, context-based interventions addressing these determinants can greatly improve women's quality of life, decrease the prevalence of UVP, and improve overall maternal health.

## Introduction

Uterovaginal prolapse (UVP) is defined as descent of the uterus through the vaginal canal due to defects in the supportive structures of the uterus and vagina because of different factors (1). Uterovaginal prolapse is a public health concern worldwide and contributes to reproductive health morbidity among women. The estimated worldwide incidence of pelvic organ prolapse is nearly 9% (1). Given the global increase in the aging population in well-resourced countries, the need for UVP management is anticipated to increase in the coming decades (2). The exact prevalence of utero vaginal prolapse is difficult to determine because many women are symptomatic, but they are not symptomatic due to social factors (3). One population-based study revealed that approximately 3% of the 1961 adult women surveyed reported symptomatic vaginal bulging (4). Globally, from 1990 to 2019, 2%–20% of all women were affected by UVP (5). In addition, the burden of UVP in low- and middle-income countries, particularly in sub-Saharan African countries, is still challenging and increasing and was recently reported to be approximately 20% from 2012 to 2015 (6). These might require different strategies to address the burden.

UVP is a common gynecological problem, and its presentation varies in type and severity among patients. In developed countries, the prevalence is high among postmenopausal women, whereas in developing countries, the condition is common in women of reproductive age (7). It has been argued that prolapse may be more common in resource-constrained settings owing to established factors and heavy physical burdens and that this condition may affect daily life more severely than it does in high-income settings (8). Working on reproductive-age groups is essential for addressing UVP in resource-limited settings.

In Ethiopia, gynecological problems are important public health problems that affect maternal health outcomes and women's productivity. In 2020, the overall incidence of UVP in Ethiopia was estimated to be 23.52% (9). Currently, UVP accounts for 40.7% of major gynecological operations in Jimma (10), followed by hysterectomy (41.1%) and leiomyoma (23%) in Tikur Anbessa (11). This underscores multi-sectoral collaboration to fight against UVP.

The true risk factors associated with UVP are poorly understood. The cause of this disorder is likely multifactorial and attributable to a combination of risk factors, which vary from patient to patient. This might be due to the private and asymptomatic nature of the illness, making UVP the “hidden epidemic” (12). The major risk factors for the development of utero-vaginal prolapse are older age, a family history of UVP, menopause, higher parity, difficult labor and delivery, malnutrition, chronic cough and constipation. Race is also considered a risk factor for prolapse, while African and Asian ethnicity is thought to be protective (13). Elucidating the determinants of UVP is important for designing context-based interventions.

Uterine prolapse is a hidden problem, especially in developing countries such as Ethiopia, where the situation is far worse (14). UVP can severely affect a woman's quality of life, with a great negative impact on women's social, physical, economic, and psychological wellbeing (15). Most importantly, problems or physical disorders that occur in patients with prolapse can reduce women's reproductive health, such as discomfort because of masses in and out of the genitals (14). Despite its burden, there are no national UVP-targeted intervention strategies. Hence, identifying the factors associated with UVP has a great role in the development of intervention strategies.

Although UVP is a preventable and curable condition, it is a prevalent disease that is impacted by various risk factors, but only a  few studies exist on its determinants. Identifying key risk factors may enable early  detection, prevention, and management measures. Thus, this study aimed to identify risk factors associated with utero vaginal prolapse (UVP) among  women attending gynecologic outpatient departments in public hospitals in the Sidama region, Ethiopia. These results provide valuable insight for policymakers, gynecologists, and midwives in the development of preventative methods, the design of patient education, and the development of treatments to minimize the burden of UVP.

## Methods and materials

### Study design

A facility-based unmatched case‒control study design was used to respond to the research objective.

### Study area and period

**Figure 1:**
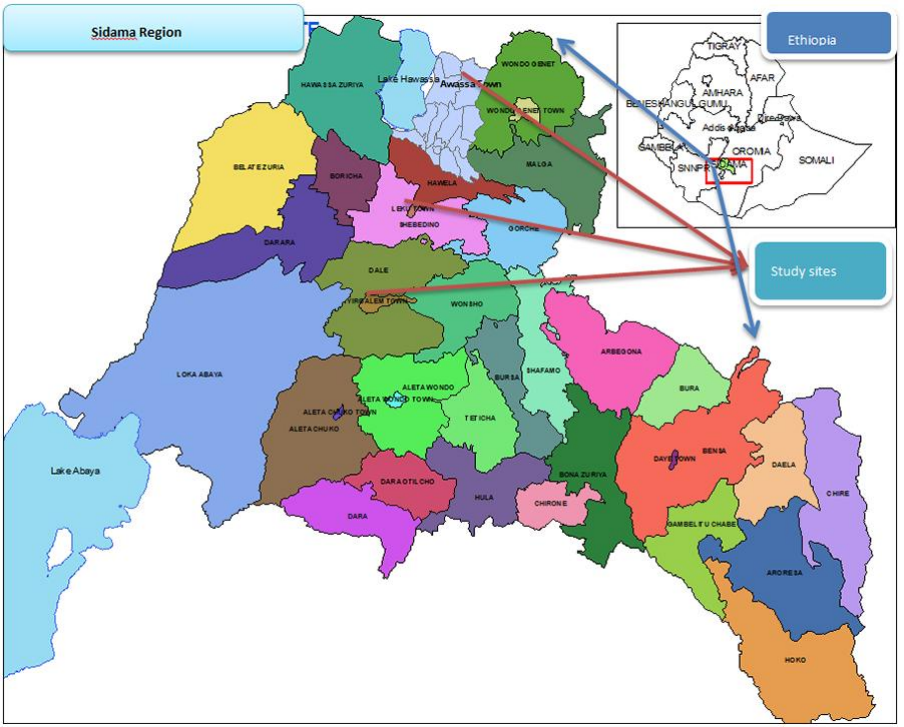
Study area administrative map.

The study was conducted in the Sidama region, which is located in the southern part of Ethiopia. The Sidama region is the tenth region in Ethiopia and is bordered by Oromia in the northeast, Wolayta in the west, and Gedo in the south. According to the Sidama Region Development Corporation, Planning and Statistics 2020, the total estimated population is 4,369,214 million people, 2,201,313 of whom are females (16). According to regional health bureau data, there are 14 primary hospitals, 4 general hospitals, 1 specialized teaching hospital, 124 governmental health centers, 526 health posts and more than 108 private clinics (17). These studies were conducted in governmental hospitals, namely, the Adare, Yirgalem, Leku and Hawassa hospitals (Figure 1), which are 275 km, 325 km, 392 km and 273 km away from Addis Ababa, respectively. The hospitals provide services for the town and surrounding area, including inpatient, outpatient, and chronic disease follow-up services and maternal and child health services. These governmental hospitals have gynecologic outpatient departments (OPDs) and gynecologic wards after they perform different gynecologic operations, including UVP. According to previous reports, the total number of UVP patients was 194, 108, 84 and 93 at Yirgalem, Hawassa Hospital, Adare and Leku hospitals, respectively (18–21). The study was conducted from May 1–July 30, 2023.

### Source and study population

#### Source populations

⮚ The source population included all women who were visiting gynecologic OPDs at government general hospitals in the Sidama region during the study period.

### Study populations

#### Case

⮚ All women older than 18 years who were attending a gynecologic OPD at a selected public hospital in Hawassa town with UVP confirmed by an attending physician during the actual data collection period were considered cases.

#### Control

⮚ All women who had no UVP but were older than 18 years and were attending selected hospitals for other gynecological care during the actual data collection period composed the study population.

### Inclusion and exclusion criteria

#### Inclusion criteria

⮚ All women older than 18 years old attending gynecology OPD during the actual data collection period in selected public health facilities were included in the study.

#### Exclusion criteria

⮚ All women who were critically ill or had a previous history of total abdominal or vaginal hysterectomy were excluded from the study.
⮚ Women with grade I UVP, women with cervical elongation, women with total abdominal hysterectomy and vaginal hysterectomy, critically ill women and women with mental problems were excluded from the study.

### Sample size determination

The sample size for the case‒control study was determined via the double population proportion formula with epi info version 7, with the following basic assumption: 95% confidence level, 80% power and a case‒to-control ratio of 1:2 (22). In a study performed in Nekemte town, East Wollega Zone, Oromia region, Ethiopia, a family history of UVP was taken as the main exposure variable for utero‒vaginal prolapse, which provided the maximum sample size (23). According to the present study, 5.3% of the controls and 20.7% of the patients had a family history of UVP. Therefore, the use of the stat calc epi info version 7.2 resulted in a total sample size of 260 patients (87 patients and 173 controls). With a 10% nonresponse rate, the total sample size on the basis of Kelsey's estimation was 286 (95 cases and 190 controls) (23, 24).

### Sampling technique and sampling procedure

A convenient sampling technique was used for enrolling patients, whereas a systemic sampling technique was used to include controls. The selected public hospitals in the Sidama region were included in the study. Prior to the study, the number of patients who attended the hospital during the past year was checked. The enumeration of the previous one-year cases and controls from the patient/client card was conducted to determine the case and control flow of each hospital with reference to their card number from the obstetric registration book. Then, on the basis of the number of patients/clients, the calculated sample size was proportionally allocated. Finally, every patient and the next two consecutive controls were included in the study until the required sample was met.

### Data collection tools and procedure

The data were collected via a pretested and structured questionnaire, which was prepared after different studies were reviewed. The questionnaire is composed of three main parts: mothers’ socio-demographic (socioeconomic) factors, gynecologic and obstetric factors, and medical and personal factors. The tool was translated into the local languages Amharic and Sidamgna to maintain consistency.

Face‒to-face interviews were used to collect the data. Previously, the final version of the questionnaire was designed with a Kobo collection kit installed on an android smartphone. The kobo tool is an easy, open electronic data collection tool suitable for field research and helps ensure data security. The data were subsequently linked to a server and checked for proper functioning. During the data collection period, 4 BSc midwives were used to collect the data, and daily supervision was provided. In addition, the principal investigator and supervisor supervised the daily data gathering in the field.

### Data quality assurance

Initially, the investigator developed the English version of the questionnaire after performing a thorough literature analysis. The questionnaire was subsequently translated into the Amharic and Sidamgna versions and then retranslated into English by an expert to ensure consistency. A pretest was conducted at Tula Hospital on 5% of the sample, and minor corrections and modifications were made before actual data collection. In addition, two days of standard training were provided to the data collectors and supervisors to familiarize them with the data collection tools. During the data gathering process, supervision was conducted, and appropriate corrective actions were taken. The collected data were checked for completeness, and any incomplete entries were excluded from the study.

### Variables of the study

#### Dependent variable

Uterovaginal prolapse.

#### Independent variables

The socioeconomic and demographic factors included place of residence, maternal age, marital status, living arrangement, maternal educational status, husband educational status, occupation, and average monthly income.

The following obstetric and gynecological factors were assessed: parity, gravidity, mode of delivery, place of delivery, duration of labor, induced labor, history of instrumental delivery, episiotomy, vaginal tear, sphincter damage, fundal pressure, ANC follow-up, prior pelvic surgery, and history of blunt/sharp damage to the reproductive or perineum.

The personal and medical factors included a history of cough, hypertension, DM, chronic constipation, a family history of UVP, and a heavy workload.

### Operational definitions

Pelvic organ prolapse refers to the abnormal herniation of pelvic viscera, such as the uterus, vaginal vault, bladder, rectum, and small or large bowel, against the vaginal walls or through the vaginal introits (6).

Uterovaginal prolapse is the descent of the uterus/cervix and vaginal segments through the vaginal canal.

Control: Women without UVP who are admitted to the hospital for other gynecological problems will be considered controls.

### Data processing and analysis

Once the data were collected, they were downloaded and exported from the kobo tool server in Excel and SPSS label forms and then imported into the Statistical Package for Social Science version 27 for analysis. The data were subsequently cleaned, followed by the identification and examination of outliers via descriptive analysis. Following the process of data cleaning, a descriptive analysis (continuous and categorical) was conducted. The results are presented via frequency tables, textual descriptions, graphs, and measures of central tendency (means) and variability (standard deviations). For continuous variables, the means and standard deviations were utilized. To assess the equality of variance and mean difference in exposure between cases and controls, we used an independent sample *t*-test. This allowed us to determine the mean difference in exposure between the two groups of people.

Then, bivariable and multivariate analyses were carried out to assess the correlations between the dependent and independent variables. Variables with *p values* less than 0.25 were candidates for multivariate analysis to account for any potential confounding effects. Finally, a *p value* <0.05 indicated a statistically significant correlation with the 95% CI and adjusted odds ratio. The Hosmer and Lemeshow goodness-of-fit test was performed to verify model fitness at a *p value* >0.05. The multi-collinearity of independent variables was tested via variance inflation factors, and a variance inflation factor (VIF) > 10 was considered suggestive of collinearity.

### Ethical considerations

Ethical approval was obtained from the research review committee of Yanet-Liyana Health Science College (Ref: LHC/YLCH/OGL/1080/15 and Date: 24/07/2023). The letter of support was subsequently obtained from the Sidama Regional Health Bureau and written to the Adare, Yirgalem, HUCSH and Leku General Hospitals. All ethical issues were addressed during data collection in the field, and written consent was obtained before actual data collection. No personal identifiers were used.

## Results

A total of 277 respondents, including 91 patients with UVP and 186 controls without a diagnosis of UVP, were included in the study, for a response rate of 97%.

### Socio-demographic factors

The mean (±SD) ages for the cases were 43.9 ± 11.6 years and 33.2 ± 10.8 years for the control group; 49 (53.3%) patients, whereas 43 (46.7%) controls, were ≥40 years old. Among the controls, 120 (80.0%) and 30 (20.0%) patients were from urban areas, respectively. Most of the controls (64, 88.9%) were from college and above. In total, 74 (52.9%) patients were house wives. Seventy-seven (52.9%) controls were married and living together. Among the married women, 55 (73.3%) of the controls had a husband with a college education or above. Additionally, 41 (67.2%) of the controls were government employees (Table 1).

**Table 1:** Socio-demographic characteristics of the respondents in the Sidama region, southern Ethiopia, 2023.

Variable	Category	Controls (%)	Cases (%)
Age	<40	143 (77.3)	42 (22.7)
	≥40	43 (46.7)	49 (53.3)
Residence	Urban	120 (80.0)	30 (20.0)
	Rural	66 (52.0)	61 (48.0)
Education level			
	Can't read and write	28 (15.1)	43 (47.3)
	Can read and write but no formal education	4 (2.2)	7 (7.7)
	Primary (grade 1–8)	41 (22)	27 (29.7)
	Secondary (grade 9–12)	49 (26.3)	6 (8.8)
	Collage and above	64 (34.4)	8 (8.8)
Occupational status	House wife	66 (47.1)	74 (52.9)
	Government employee	39 (84.8)	7 (15.2)
	Private worker	39 (90.7)	4 (9.3)
	Unemployed	25 (100.0)	0 (0.0)
	Merchant	17 (73.9)	6 (26.1)
Marital status	Married and living together	77 (52.0)	71 (48.0)
	Married but not living together	30 (88.2)	4 (11.8)
	Never married	56 (100.0)	0 (0.0)
	Widowed	15 (57.7)	11 (42.3)
	Divorced	8 (61.5)	5 (38.5)
Husband education level	Can't read and write	22 (36.1)	39 (63.9)
	Non formal education	8 (66.7)	4 (33.3)
	Primary	19 (50.0)	19 (50.0)
	Secondary	26 (74.3)	9 (25.7)
	College and above	55 (73.3)	20 (26.7)
Husband occupation level	Government employee	41 (67.2)	20 (32.8)
	Private worker	22 (71.0)	9 (29.0)
	Farmer	31 (38.8)	49 (61.3)
	Merchant	1 (100.0)	0 (0.0)
	Unemployed	36 (73.5)	13 (26.5)

### Obstetric and gynecologic factors

Among the study respondents, 153 (80.5%) were controls, and 37 (19.5%) were married at the age of 18 and above. Among the respondents, 70 (71.4%) controls and 28 (28.6%) patients gave their first birth at the age of 20 years and above. Among the patients, 50 (35.2%) had fewer than 5 live births, and 44 (33.1%) had fewer than 5 pregnancies. The majority (80, 65.6%) of the controls had ANC visits. More than one-fourth of the patients (80; 54.1%) delivered vaginally. More than half of the patients (58.0%) had a history of multiple pregnancies. Among the respondents, 23 (43.4%) were controls, and 30 (56.6%) gave birth at home. Among the patients, 37 (52.9%) had a history of labor greater than or equal to 24 hours. Among the patients, 9 (25.7%), 23 (43.4%) and 8 (29.6%) had a history of instrumental delivery, episiotomy and induced labor, respectively. Among the patients, 12 (27.3%), 3 (27.3%) and 39 (59.1%) had a history of vaginal tear, sphincter damage and fundal pressure, respectively (Table 2).

**Table 2:** Gynaecologic and obstetric characteristics of the respondents from governmental health facilities in Sidama region, southern Ethiopia, 2023.

Variables	Category	Controls (%)	Cases (%)
Age at first marriage	<18	33 (37.9)	54 (62.1)
	≥18	153 (80.5)	37 (19.5)
Age at first child birth	<20	39 (3.2)	63 (61.8)
	≥20	70 (71.4)	28 (28.6)
Number of birth	<5	92 (64.8)	50 (35.2)
	≥5	17 (29.3)	41 (70.7)
Number of pregnancy	<5	89 (66.9)	44 (33.1)
	≥5	20 (29.9)	47 (70.1)
ANC utilization	Yes	80 (65.6)	42 (34.4)
	No	29 (37.2)	49 (62.8)
Delivery mode	SVD	68 (45.9)	80 (54.1)
	CS	38 (82.6)	8 (17.4)
	Instrumental	3 (50.0)	3 (50.0)
Multiple pregnancy(history)	Yes	42 (42.0)	58 (58.0)
	No	67 (67.0)	33 (33.0)
Place of last child birth	Home	23 (43.4)	30 (56.6)
	Health institution	86 (58.5)	61 (41.5)
Labour >24 h	Yes	33 (47.1)	37 (52.9)
	No	76 (58.5)	54 (41.5)
Instrumental delivery(history)	Yes	26 (74.3)	9 (25.7)
	No	83 (50.3)	82 (49.7)
Episiotomy(history)	Yes	30 (56.6)	23 (43.4)
	No	79 (53.7)	68 (46.3)
Induced labour(history)	Yes	19 (70.4)	8 (29.6)
	No	90 (52.0)	83 (48.0)
Vaginal tear(history)	Yes	32 (72.7)	12 (27.3)
	No	77 (49.4)	79 (50.6)
Sphincter damage(history)	Yes	8 (72.7)	3 (27.3)
	No	101 (53.4)	88 (46.6)
Fundal pressure(history)	Yes	27 (40.9)	39 (59.1)
	No	82 (61.2)	52 (38.8)

Among the study respondents, 181 (65.3%) had postpartum rest before starting their usual activity. Among these respondents, 57 (48.3%) patients and 61 (51.7%) controls had less than 42 days of rest.

### Medical factors

Among the women involved in the study, 33 (66.0%) controls and 17 (34.0%) patients had a history of chronic cough. Twenty-four (55.8%) controls and 19 (44.2%) patients had chronic constipation. Three (16.7%) and 10 (28.6%) patients had diabetes mellitus and hypertension, respectively.

### Individual factors

Among the study respondents, 28 (53.8%) controls and 24 (46.2%) patients had a history of abortion. Among the total patients, 5 (16.1%) and 4 (16.7%) had undergone prior pelvic surgery and reconstruction organ blunt surgery, respectively. Information about the cause of UVP was available for 50 (72.5%) controls and 19 (27.5%) patients. Information about the cause of UVP was available for 35 (87.5%) controls and 5 (12.5%) patients. There were 29 (87.9%) controls and 4 (12.1%) patients with information about aggravating factors of UVP. Among the respondents, 20 (83.3%) controls and 4 (16.7%) patients had a family history of UVP. Among the women involved in the study, 142 (78.0%) controls and 40 (22.0%) patients had a history of heavy objects.

**Figure 2:**
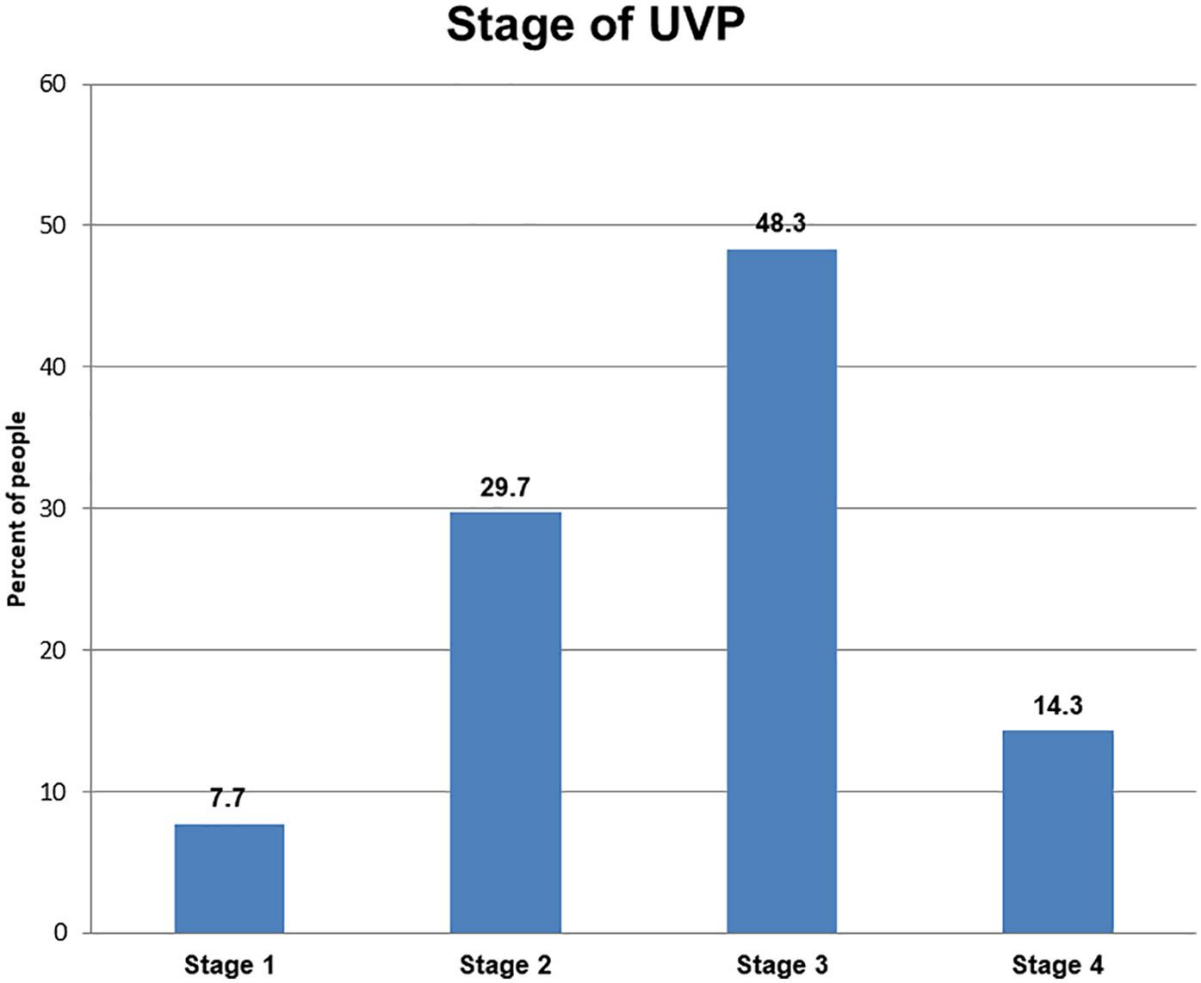
Stages of UVP at governmental hospitals in the Sidama region, 2023.

Among all UVP patients, 44 (48.3%) were diagnosed with stage 3 UVP, followed by 29.7% who were diagnosed with stage 2 UVP (Figure 2).

### Independent sample *t*-tests for the mean difference in exposure between cases and controls

To assess the equality of variance and mean difference between cases and controls with respect to exposure factors, in this study, we performed independent sample *t*-tests. Levene's test for the equality of variances was applied that is, equal variances, *p* > 0.05, and unequal variances, *p* < 0.05 to investigate the similarity of variance between cases and controls. Similarly, the mean difference between the case and control groups was determined via a *t*-test for equality, with corresponding *p* values applied (i.e., *p* < 0.05 signifies a significant variation in the means of the two sample groups evaluated). We assumed unequal variance and applied the one-sample *t*-test, as practically all of the test variables have a variance greater than 4 (25). The factors associated with increased risk of UVP among women were highest educational level (*P* < 0.0001), respondent marital status (*P* < 0.001), multiple pregnancies (*P* < 0.001), history of home delivery (*P* < 0.001), history of instrumental delivery (*P* = 0.008), history of fundal pressure during childbirth (*P* = 0.007), and farming experience (*P* < 0.001) (Table 3).

**Table 3:** Independent sample *t*-test of respondents for determinants of UVP among women visiting gynaecologic outpatient departments at governmental hospitals in the Sidama region, southern Ethiopia, 2023 (cases = 95, control = 190; total 286.

Independent samples test										
Variables		Levene's test for equality of variances		*t*-test for equality of means						
		F	Sig.	t	df	Sig. (2-tailed)	mean difference	Std. error difference	95% confidence interval of the difference	
									Lower	Upper
Place of residence	Equal variances assumed	.716	.398	5.165	275	.000	.315	.061	.195	.436
	Equal variances not assumed			5.192	181.185	.000	.315	.061	.196	.435
Highest educational level	Equal variances assumed	.273	.602	−8.097	275	.000	−1.409	.174	−1.752	−1.067
	Equal variances not assumed			−8.160	182.426	.000	−1.409	.173	−1.750	−1.068
Respondent occupational status	Equal variances assumed	26.192	.000	−6.053	275	.000	−.969	.160	−1.285	−.654
	Equal variances not assumed			−6.529	218.181	.000	−.969	.148	−1.262	−.677
Respondent marital status	Equal variances assumed	.414	.521	−3.543	275	.000	−.551	.156	−.857	−.245
	Equal variances not assumed			−3.450	166.917	.001	−.551	.160	−.866	−.236
husband's highest educational level	Equal variances assumed	3.536	.061	−4.996	219	.000	−1.055	.211	−1.471	−.639
	Equal variances not assumed			−4.920	182.919	.000	−1.055	.214	−1.478	−.632
Respondent give birth	Equal variances assumed	2,961.908	.000	−7.989	275	.000	−.414	.052	−.516	−.312
	Equal variances not assumed			−11.432	185.000	.000	−.414	.036	−.485	−.343
Mode your last delivery	Equal variances assumed	33.840	.000	−3.498	198	.001	−.250	.071	−.391	−.109
	Equal variances not assumed			−3.563	197.896	.000	−.250	.070	−.388	−.112
Multiple pregnancies	Equal variances assumed	.436	.510	−3.649	198	.000	−.252		−.388	−.116
	Equal variances not assumed			−3.653	192.413	.000	−.252	.069.069	−.388	−.116
History of home delivery	Equal variances assumed	.020	.888	−4.081	198	.000	−.280	.069	−.415	−.144
	Equal variances not assumed			−4.080	191.414	.000	−.280	.069	−.415	−.144
History of instrumental delivery	Equal variances assumed	31.618	.000	2.619	198	.009	.140	.053	.035	.245
	Equal variances not assumed			2.701	192.526	.008	.140	.052	.038	.242
History of vaginal tear during child birth	Equal variances assumed			2.789	198	.006	.162	.058	.047	.276
	Equal variances not assumed	35.692	.000	2.862	195.519	.005	.162	.057	.050	.273
History of fundal pressure during child birth	Equal variances assumed	23.932	.000	−2.746	198	.007	−.181	.066	−.311	−.051
	Equal variances not assumed			−2.712	180.010	.007	−.181	.067	−.312	−.049
Information about the cause of utero vaginal prolapse	Equal variances assumed	46.194	.000	3.000	275	.003	.133	.044	.046	.221
	Equal variances not assumed			3.557	266.452	.000	.133	.037	.059	.207
Farming experienced	Equal variances assumed	85.080	.000	−6.373	275	.000	−.785	.123	−1.027	−.542
	Equal variances not assumed			−5.457	124.703	.000	−.785	.144	−1.069	−.500
Involved in wood collection	Equal variances assumed	75.364	.000	−7.267	275	.000	−.946	.130	−1.202	−.690
	Equal variances not assumed			−6.309	128.211	.000	−.946	.150	−1.243	−.649
Involved in fetching water	Equal variances assumed	30.488	.000	−6.266	275	.000	−.801	.128	−1.053	−.549
	Equal variances not assumed			−5.745	144.369	.000	−.801	.139	−1.077	−.525

### Determinants of utero-vaginal prolapse

In the bivariate logistic regression analysis, age, residence, education, husband education level, age at child birth, number of births, history of instrumental delivery, number of pregnancies, ANC visit, multiple pregnancies, history of induced labor, prior pelvic surgery, chronic constipation, information about UVP, place of delivery and workload were found to be variables selected for multivariate analysis. On multivariate analysis, age at first childbirth, multiple pregnancies, place of delivery, prior pelvic surgery and history of instrumental delivery were found to be potential determinants of UVP at a *p* value <0.05.

The odds of developing UVP were 4 times greater among women aged less than 20 years with a first child birth. [AOR = 3.98 (95% CI: 1.1–14.6)]. Women who had a history of multiple pregnancies were approximately three times more likely to develop UVP than their male counterparts were. [AOR = 2.8 (95% CI: 1.3–6.5)]. Women who delivered at home were 5 times more likely to develop UVP than those who delivered at health institutions were [AOR = 4.9 (95% CI: 1.3–18.6)]. Women who had prior pelvic surgery were 4 times more likely to develop this condition than their counterparts were. [AOR = 3.9 (95% CI: 1.1–13.9)]. Women who had a history of instrumental delivery were approximately three times more likely to develop UVP than their counterparts were. [AOR = 3.1 (95% CI: 1.1–9.11)] (Table 4).

**Table 4:** Multivariable and bivariable characteristics on determinates of UVP in governmental hospitals in the Sidama region, southern Ethiopia, 2023.

Variable	Controls	Cases	COR (95% CI)	*P* value	AOR (95% CI)	*P* value
Age						
<40	143	42	1		1	
≥40	43	49	3.8 (2.3–6.6)	0.001	1.1 (0.4–2.7)	0.843
Residence						
Urban	120	30	1		1	0.302
Rural	66	61	0.3 (0.2–0.5)	0.001	1.7 (0.6–4.4)	
Education						
Can't read and write	28	43	12.3 (5.1–29.5)	0.001	0.8 (0.1–5.4)	0.853
Non formal education	4	7	14.0 (3.3–58.6)	0.001	0.8 (0.1–5-6.5)	0.858
Primary	41	27	5.3 (2.2–12.7)	0.001	1.3 (0.4–4.6)	0.646
Secondary	49	6	0.9 (0.3–3.0)	0.971	0.7 (0.2–2.9)	0.635
College and above	64	8	1		1	
Husband education						
Can't read and write	22	39	4.2 (2.1–8.5)	0.001	0.4 (0.1–3.2)	0.376
Non formal education	8	4	0.4 (0.1–1.2)	0.091	2.0 (0.4–10.0)	0.398
Primary	19	19	1.8 (0.8–3.8)	0.126	0.4 (0.1–2.4)	0.312
Secondary	26	9	0.9 (0.3–2.1)	0.737	0.5 (0.1–3.0)	0.439
College and above	55	20	1		1	
Age at first child birth						
<20	39	63	4.0 (2.2–7.3)	0.001	3.9 (1.1–14.6)^a^	0.037^a^
≥20	70	28	1		1	
Number of birth						
<5	92	50	1		1	
≥5	17	41	4.4 (2.3–8.6)	0.001	1.5 (0.2–11.3)	0.675
Number of pregnancy						
<5	89	44	1		1	
≥5	20	47	4.7 (2.5–8.9)	0.001	4.8 (0.7–31.8)	0.104
ANC utilization						
Yes	80	42	1		1	
No	29	49	3.3 (1.7–5.8)	0.001	1.9 (0.6–6.6)	0.274
Multiple pregnancy						
Yes	42	58	2.8 (1.6–4.9)	0.001	2.8 (1.3–6.5)^a^	0.011^a^
No	67	33	1		1	
Place of delivery						
Home	23	30	1.8 (0.9–3.5)	0.001	4.9 (1.3–18.6)^a^	0.018^a^
Health institution	86	66	1		1	
Instrumental delivery						
Yes	26	9	2.8 (1.3–6.5)	0.012	3.1 (1.1–9.1)^a^	0.036^a^
No	83	82	1		1	
Induced labour						
Yes	19	8	2.2 (0.9–5.3)	0.080	1.5 (1.1–1.6)	0.224
No	90	83	1		1	
Prior pelvic surgery						
Yes	26	5	2.8 (1.0–7.5)	0.042	3.9 (1.1–13.9)^a^	0.038^a^
No	160	86	1		1	
Chronic constipation						
Yes	24	19	1.8 (0.9–3.5)	0.088	1.7 (1.3–2.1)	0.613
No	162	72	1		1	
Information about UVP						
Yes	50	19	0.3 (0.0–0.7)	0.005	1.3 (0.3–5.4)	0.705
No	136	72	1		1	
Work load						
Light	44	51	1		1	
Heavy	142	40	4.1 (2.4–7.0)	0.001	1.0 (0.4–2.6)	0.944

NB^a^: Variables that have a significant association according to multivariable logistic regression analysis, 1: Means reference category.

## Discussion

This study highlights the key determinants of utero-vaginal prolapse among women visiting gynecology outpatient departments in governmental hospitals in the Sidama region of Ethiopia. After controlling for potential confounders, age at first birth, history of multiple pregnancies, place of delivery, prior pelvic surgery and history of instrumental delivery were found to be potential determinants of UVP.

The findings of this study revealed that women aged less than 20 years with a first child had a 3.9-fold greater chance of developing UVP than their counterparts did. This finding is in agreement with findings from a public referral hospital in the Amhara region and Nepal (26, 27). This might be because the supportive ligament may not mature and may not be strong enough to avoid prolapse of the pelvic muscle below the age of 20. Additionally, findings from Kathmandu, Nepal, revealed that having a child under the age of 20 years was associated with the development of UVP; accordingly, the study cited a possible reason for poor decisions related to personal well-being. Because of poor decisions, women under the age of 20 are unable to discontinue uterine prolapse risk behaviors (28). The possible justification could be that child birth through the vagina at a young age may involve prolonged or obstructed labor that may injure the pelvic floor fascia and muscles.

In this study, women who had a history of multiple pregnancies were 2.8 times more likely to develop UVP than their counterparts were. This finding is congruent with other studies from Bahir Dar, Southeast Nigeria, Pakistan and Turkey (29–32). Another study conducted to assess the morphological characteristics of the pelvic floor musculature between women with twin pregnancies and those with singleton pregnancies revealed that pelvic support undergoes greater changes during twin pregnancy (33). Multiple pregnancies or twin pregnancies can damage the sphincter muscles and ligaments. The possible justification could be that multiple pregnancies may face repeated cycles of stretching and straining during labor to the pelvic floor, which may lead to muscle tone and ligament weakening.

This study revealed that women who delivered at home were 4.9 times more likely to develop UVP than those who delivered at health institutions were. This finding is in line with other findings from Southwest Ethiopia, Wolayta Sodo, South Ethiopia, Tanzania and Nepali (34–37). This finding was also supported by a pooled analysis conducted in Ethiopia, where home delivery resulted in the development of UVP due to a greater risk of prolonged labor and perinatal tears (38). Prolonged labor is a well-established cause of UVP. When the fetal head applies pressure to the pelvic floor for prolonged periods during its engagement in the birth cannula, the pelvic floor muscle, tissue, nerve, and other supporting structures are damaged. This condition may result in the downward displacement of the pelvic organs from their normal position (39). Another possible reason is that home delivery by unskilled attendants causes significant damage to the pelvic support system. This condition may also result in UVP as a long-term complication (27). The possible reasons could be recently home delivery conducted in Ethiopia without skilled birth attends that usually services provided by traditional birth attends or grandmother that may cause improper management of labour that can lead prolong labour/pushing greatly, overstretch and damage in pelvic floor fascia, nerves and muscles. In health facility settings typically employ continuous fetal monitoring, have immediate access to obstetric interventions, and often follow time-bound labor management protocols. In contrast, home delivery may allow for more neglected labor management and may take long durations and less intervention approaches. These factors could influence not only maternal perceptions of the birth experience but also the physiological outcomes observed.

In this study, women who had prior pelvic surgery were 3.9 times more likely to develop UVP. This finding is consistent with findings from Asella Teaching and References Hospital and England (40, 41). Another study from the American College of Obstetricians and Gynecologists revealed that women who underwent primary UVP surgery had an approximately 30%–50% chance of needing a second prolapse surgery (42). This might be because of a high likelihood of pelvic floor muscle injury during surgical procedures. The other reason could be that previous pelvic surgery may weaken ligaments and alter the structures that may reduce pelvic floor strength.

The odds of having UVP were 3.1 times greater among those with a history of instrumental delivery than among their counterparts. These findings were supported by a study conducted in Nekemte, Western Ethiopia (23). This was explained by the fact that forceps delivery is associated with muscle trauma, which results in pelvic floor muscle damage (38). However, this finding was not supported by a meta-analysis conducted in Ethiopia, which revealed no significant difference in UVP between assisted vaginal delivery (including vacuum and forceps) and spontaneous vaginal delivery (43). This was because, according to the pooled estimates, no significant association was found between vacuum delivery and primary UVP (43, 44). The reason could be that prolonged labor pushing, which may precede forceps delivery, may weaken tissues and may cause compression and traction during the prolonged second stage and instrumental extraction, which may lead to trauma to the pelvic floor muscle and fascia.

### Implications of the study

The study of risk factors for utero-vaginal prolapse (UVP) among women visiting gynecologic outpatient departments in the Sidama region provides crucial insights into risk factors and potential intervention points for addressing this public health issue. The key implications of these findings are as follows: public health significance implications for women in the early age group and women with multiple births identified as risk factors. We hope these findings provide insight into the design of tailored interventions for most vulnerable groups. In addition, our findings demonstrated that strengthening early screening to detect risk factors is critical to address this problem, so it has clinical practice implications. Furthermore, our findings suggest that strengthening maternal health and obstetric care, such as skilled delivery services, to prevent home delivery and promoting the use of contraceptives to reduce high parity may lead to a reduction in the burden of UVP. These findings underscore policy and program implications. In addition, policymakers should prioritize health education campaigns targeting women and communities to increase awareness of risk factors for uterovaginal prolapse. Therefore, designing tailored interventions for at-risk groups is a nondoubtful action to enhance maternal health and well-being.

### Strengths and limitations of the study

In this study, researchers included multiple health facilities, which could help to obtain adequate sample sizes. This study has various limitations, such as the fact that some obstetric characteristics were reported by patients who may have long durations since the patients were asked about their previous history, which may have led to recall bias. Moreover, the study was conducted at a hospital-based scale and did not include a specific population or community; therefore, it may be difficult to generalize the findings to the entire community. In addition, the study did not assess nutritional status.

## Conclusion

The current study highlights that pertinent risk factors, such as socio-demographic, obstetric & gynecologic and medical and personal factors, were identified as key determinants of UVP. These findings may require the attention of policy makers to alleviate this problem. Moreover, health professionals should counsel about the consequences and complications of early childbirth, multiple pregnancies, instrumental delivery and the effects of home delivery and their associations with UVP development. In addition, prevention strategies such as pelvic floor muscle training and elective cesarean section for women who are at high risk are recommended to address this problem.

We would like to express our deepest gratitude to Yanet-Liyana College of Health Sciences, Department of Reproductive Health, for providing ethical clearance to conduct this study. Our deepest gratitude goes to Adare, Yirgalem, HUCSH and Leku General Hospital for their support and facilitation during our data collection. We appreciate the study participants who voluntarily participated in the study. We acknowledge our data collectors and supervisors who took part in data collection in the field.

## Data Availability

The original contributions presented in the study are included in the article/Supplementary Material, further inquiries can be directed to the corresponding author.
